# Parliament and the Profession

**Published:** 1916-10-28

**Authors:** 


### PARLIAMENT AND THE PROFESSION.

### Dentists in Mesopotamia.

IN answer to a question from Sir John Jardine, Mr. Lloyd George stated that the Force in Mesopotamia had been supplied with all the dentists and dentists' mechanics asked for.

### Doctor's Motor-Boat.

THE Secretary to the Admiralty informed Mr. Wason that a doctor who, by terms of his contract, has to keep a sea-going motor-boat is bound to comply with any directions given by a competent authority in respect of the use of land lights.

### Glare Goggles in Mesopotamia.

MR. LLOYD GEORGE informed Major Hunt that an ample supply of goggles of good pattern has been dispatched to the troops in Mesopotamia, and that so far as the Army Council is aware it is not a fact that these goggles had been found useless.

### Statistics of Medically Unfit Men.

MR. LLOYD GEORGE, in answer to an inquiry on the part of Mr. Jacobsen, said that no statistics are available as to men passed into the Army and discharged as medically unfit within three months, and he did not see his way to burden the Record Office with the heavy task of providing the information.

### Vaccination and Inoculation.

MR. FORSTER said, in reply to Mr. Snowden, that it was not proposed to take any action in respect of certain soldiers who had been stopped leave because they had not submitted to vaccination and inoculation; and, in answer to Mr. Thorne, that he was not prepared to intervene in any action taken by the British Commander-in-Chief in France to preserve the health of the troops under his command.

### Hospital Authorities and War Pensions.

A NUMBER of questions were put to Mr. Forster to ascertain what steps are being taken to expedite the consideration of applications for pensions. A suggestion was made that hospital authorities should notify local Pension Committees as to discharges, Mr. Forster said that although he was reluctant to throw any additional work on the hospital authorities, he would consider what could be done in the matter.

### Medical Men as Combatant Officers.

MR. FORSTER, in reply to a question put by Mr. Perkins as to the position of medical men who are serving as combatant officers in respect of rates of pay and length of service, stated that qualified medical men serving as combatant officers in the Territorial Force had been given an opportunity of serving in the Royal Army Medical Corps. Mr. Forster said he would be pleased to make inquiries into any case of hardship.

### Use of Stimulants in Military Hospitals.

THE fact that expenditure on stimulants had been greatly reduced at Guy's Hospital and Leeds Infirmary was introduced into a question put by Major Chapple to the Secretary of State for War with a view to inquiring if the military hospitals could consider similar steps. Mr. Forster replied that economy in the issue of stimulants was included in a reference to a Special Committee, under the presidency of Major Godfrey Collins, M.P., and that the amount of alcohol is limited to what is medicinally necessary.

### Discharged Sailors and Soldiers.

MR. ROBERTS, representing the National Health Insurance Commissioners, informed Mr. Nugent that in Great Britain discharged sailors and soldiers who are insured persons receive medical benefit under the ordinary arrangements. As regards Ireland, the question is being carefully considered by the Statutory Committee on Pensions.

The President of the Local Government Board answered questions of Mr. Hogge and Mr. Percy Harris as to after medical care of discharged disabled sailors and soldiers. Mr. Hayes Fisher said that the financial aspect of this matter had yet to receive the assent of the Treasury. This scheme covers Great Britain, and other arrangements are in progress with regard to Ireland. Before the scope of the scheme can be settled or its operation commenced the decision of the War Office as to the extent of the use of military hospitals must be ascertained, and the full co-operation of that Department and of the Admiralty must be secured.

